# Extracellular Vesicles: A Novel Tool Facilitating Personalized Medicine and Pharmacogenomics in Oncology

**DOI:** 10.3389/fphar.2021.671298

**Published:** 2021-04-30

**Authors:** Katja Goričar, Vita Dolžan, Metka Lenassi

**Affiliations:** Institute of Biochemistry and Molecular Genetics, Faculty of Medicine, University of Ljubljana, Ljubljana, Slovenia

**Keywords:** extracellular vesicle, pharmacogenomics, liquid biopsy, cancer, biomarker, personalized medicine

## Abstract

Biomarkers that can guide cancer therapy based on patients’ individual cancer molecular signature can enable a more effective treatment with fewer adverse events. Data on actionable somatic mutations and germline genetic variants, studied by personalized medicine and pharmacogenomics, can be obtained from tumor tissue or blood samples. As tissue biopsy cannot reflect the heterogeneity of the tumor or its temporal changes, liquid biopsy is a promising alternative approach. In recent years, extracellular vesicles (EVs) have emerged as a potential source of biomarkers in liquid biopsy. EVs are a heterogeneous population of membrane bound particles, which are released from all cells and accumulate into body fluids. They contain various proteins, lipids, nucleic acids (miRNA, mRNA, and DNA) and metabolites. In cancer, EV biomolecular composition and concentration are changed. Tumor EVs can promote the remodeling of the tumor microenvironment and pre-metastatic niche formation, and contribute to transfer of oncogenic potential or drug resistance during chemotherapy. This makes them a promising source of minimally invasive biomarkers. A limited number of clinical studies investigated EVs to monitor cancer progression, tumor evolution or drug resistance and several putative EV-bound protein and RNA biomarkers were identified. This review is focused on EVs as novel biomarker source for personalized medicine and pharmacogenomics in oncology. As several pharmacogenes and genes associated with targeted therapy, chemotherapy or hormonal therapy were already detected in EVs, they might be used for fine-tuning personalized cancer treatment.

## Introduction

Advances in the field of genomics, proteomics, and other high-throughput methods for biomarker determination have enabled the era of **personalized medicine**. Biomarkers that can guide and customize therapy for individual patients can enable a more effective treatment with fewer adverse events (Zhang J. et al., 2018). One of the biggest breakthroughs in personalized medicine was in the field of oncology, as several actionable tumor somatic mutations that enabled targeted treatment and precision medicine were identified (Table 1) (Friedman et al., 2015; Syn et al., 2016; Panagiotara et al., 2017). In **precision medicine**, genomic approaches help the clinician to select the most appropriate treatment based on the individual cancer molecular signature (Panagiotara et al., 2017). For example, patient stratification based on actionable somatic mutations is already required before the initiation of some targeted treatments (Bonanno, 2013; Syn et al., 2016). Additionally, germline genetic variants are often associated with interindividual variability in treatment response and occurrence of adverse events (Sim et al., 2013). The field of **pharmacogenomics** focuses on genetic variability of genes involved in drug metabolism or transport, drug target genes and DNA repair genes that can affect response to various drugs, including chemotherapy and hormonal therapy and thus contribute to personalized medicine. Even though several drug prescribing recommendations are available, only a handful of pharmacogenetic tests are already required before the initiation of therapy in clinical practice (Lauschke et al., 2017). Novel biomarkers that would enable additional patient stratification, continuous disease monitoring or assessment of prognosis and thus improve treatment outcome are therefore still widely investigated.

**TABLE 1:** List of pharmacogenes associated with targeted cancer therapy and the evidence connecting them with extracellular vesicles (EVs).

Gene	Drug	Connection with extracellular vesicles: Examples	References^a^
*ABL1*	Imatinib, bosutinib, dasatinib, nilotinib, ponatinib, vincristine	mRNA detected in various cancer cell EVs	Skog et al. (2008), Hong et al. (2009), Chacon-Heszele et al. (2014), Zhu et al. (2014), He et al. (2015), Fu et al. (2017), Milani et al. (2017), Kang et al. (2018)
		Protein detected in urine EVs (healthy donors)	
		BCR-ABL1 mRNA and protein detected in EVs from CML cells and in serum EVs of patients with CML	
*ALK*	Alectinib, atezolizumab, brigatinib, ceritinib, crizotinib, lorlatinib, pembrolizumab, ramucirumab	Protein detected in various cancer cell line EVs and in urine EVs (healthy donors)	Gonzales et al. (2009), Hong et al. (2009), Hurwitz et al. (2016), Wu et al. (2018), Reclusa et al. (2019)
		mRNA detected in colorectal cancer cell EVs	
		EML4-ALK translocation detected in plasma EVs in NSCLC	
*BCR*	Imatinib, bosutinib, dasatinib, nilotinib, ponatinib, vincristine	Protein detected in various cancer cell line EVs and in urine EVs (healthy donors)	Skog et al. (2008), Gonzales et al. (2009), Hong et al. (2009), Zhu et al. (2014), Hurwitz et al. (2016), Fu et al. (2017), Liem et al. (2017), Milani et al. (2017), Kang et al. (2018)
		mRNA detected in colorectal cancer and glioblastoma cell EVs	
		BCR-ABL1 mRNA and protein detected in EVs from CML cells and in serum EVs of patients with CML	
*BRAF*	Binimetinib, cobimetinib, dabrafenib, encorafenib, trametinib, vemurafenib	mRNA detected in colorectal cancer cell EVs, melanoma plasma and lymphatic drainage EVs and glioblastoma plasma EVs	Hong et al. (2009), Hao et al. (2017), Klump et al. (2018), García-Silva et al. (2019), Pasqualetti et al. (2019), Song et al. (2019)
		DNA detected in melanoma serum EVs and lung adenocarcinoma pleural-effusion EVs	
*EGFR*	Afatinib, atezolizumab, cetuximab, dacomitinib, erlotinib, gefitinib, osimertinib, panitumumab, pembrolizumab, ramucirumab	DNA detected in EVs from plasma, cerebrospinal fluid, bronchoalveolar lavage or pleural effusion of lung adenocarcinoma, NSCLC or glioblastoma patients mRNA detected in EVs from plasma or cerebrospinal fluid of NSCLC or glioblastoma patients	Skog et al. (2008), Hong et al. (2009), Welton et al. (2010), Adamczyk et al. (2011), Shao et al. (2012), Liang et al. (2013), Yamashita et al. (2013), Sinha et al. (2014), Hurwitz et al. (2016), Figueroa et al. (2017), Liem et al. (2017), Lee Y. T. et al. (2018), Castellanos-Rizaldos et al. (2018), Choi et al. (2018), Krug et al. (2018), Wan et al. (2018), Ortega et al. (2019), Qu et al. (2019), Song et al. (2019)
		Protein detected in various cancer cell line EVs and plasma EVs of breast cancer patients	
*ERBB2*	Abemaciclib, alpelisib, everolimus, fulvestrant, lapatinib, neratinib, olaparib, palbociclib, pertuzumab, ribociclib, talazoparib, tipiracil/fluridine, trastuzumab	Protein detected in various cancer cell line EVs and plasma derived EVs in gastric and breast cancer patients	Skog et al. (2008), Hong et al. (2009), Baran et al. (2010), Ciravolo et al. (2012), Liang et al. (2013), Sinha et al. (2014), Gerratana et al. (2015), Hurwitz et al. (2016), Fang et al. (2017), Liem et al. (2017), Lee J. S. et al. (2018), Choi et al. (2018), Platko et al. (2019)
		mRNA detected in colorectal cancer and glioblastoma cell EVs and plasma derived EVs in gastric and breast cancer patients	
		DNA amplification detected in urine EVs (urothelial bladder carcinoma)	
*KIT*	Imatinib	Protein detected in various cancer cell line EVs	Atay et al. (2014), Hurwitz et al. (2016)
*KRAS*	Cetuximab, panitumumab, tipiracil/fluridine	Protein detected in various cancer cell line EVs, urine EVs (healthy donors) and EVs from pleural effusions of lung cancer patients	Gonzales et al. (2009), Hong et al. (2009), Demory Beckler et al. (2013), Fraser et al. (2013), Liang et al. (2013), Park et al. (2013), Kahlert et al. (2014), Lee et al. (2014), Sinha et al. (2014), Hurwitz et al. (2016), Kamerkar et al. (2017), Liem et al. (2017), Yang S.-J. et al. (2017), Choi et al. (2018), Möhrmann et al. (2018)
		mRNA detected in colorectal cancer cell EVs	
		DNA detected in EVs from plasma or serum of pancreatic cancer patients, NSCLC, advanced cancers	
*NRAS*	Cetuximab, panitumumab	Protein detected in various cancer cell line EVs and in urine EVs (healthy donors)	Gonzales et al. (2009), Hong et al. (2009), Sinha et al. (2014), Hurwitz et al. (2016), Liem et al. (2017), Choi et al. (2018), Liu et al. (2020)
		mRNA detected in colorectal cancer cell EVs	

a References from individual functional/biomarker EV studies and EV-omics studies from VesiclePedia or Exocarta.

CML, chronic myeloid leukemia; NSCLC, non-small cell lung cancer.

Conventionally, cancer prognostic or predictive biomarkers are mostly determined in tumor tissue samples (Crowley et al., 2013). However, cancer is a very complex and heterogeneous disease, with a lot of factors contributing to its initiation, progression, development of metastasis or resistance to treatment (Zhang J. et al., 2018). Treatment resistance is not only associated with inherent genetic or non-genetic factors, but can also be acquired during or after treatment (Gong et al., 2012; Friedman et al., 2015). Tumor phenotype is therefore heterogeneous and constantly changing and can include different molecular mechanisms of carcinogenesis (Gong et al., 2012). With traditional tissue biopsy, only a specific section of the tumor is analyzed, therefore it does not reflect spatial tumor heterogeneity and patients may be stratified based on incomplete data (Crowley et al., 2013; Buder et al., 2016; Panagiotara et al., 2017). During the course of the treatment, new mutations can occur or accumulate, which can affect treatment selection or adjustment. As tissue biopsy is usually performed only at one time-point, these results are static and do not reflect the temporal changes of the tumor landscape (Crowley et al., 2013; Panagiotara et al., 2017). Tissue biopsies also have several other limitations: they are invasive and can even lead to complications. Enough material for all analyses cannot always be obtained, and some tumors are not accessible at all (Crowley et al., 2013; Buder et al., 2016; Panagiotara et al., 2017). In recent years, circulating tumor biomarkers are therefore extensively explored as an alternative minimally invasive approach called liquid biopsy (Mari et al., 2019).

A liquid biopsy is a test performed on biofluid samples to detect cancer cells or cancer-derived molecules (Vasconcelos et al., 2019). During the formation and growth of the tumor, various components may be released into the body fluids due to apoptosis, necrosis, or active release. These include circulating tumor cells (CTCs), circulating tumor DNA (ctDNA), circulating tumor RNA (ctRNA) and extracellular vesicles (EVs) (Alimirzaie et al., 2019; Vasconcelos et al., 2019). For example, ctRNA consists of mRNAs, miRNAs and long-noncoding RNAs and can be found either in ribonucleoprotein complexes, CTCs or EVs (Alimirzaie et al., 2019). All components from cancer tissue accumulate in body fluids and can be detected using liquid biopsies that reflect the genetic landscape of the whole cancer tissue. The possibility of minimally invasive serial sampling enables longitudinal monitoring of the disease at different time points. Liquid biopsy can therefore be used for screening or early diagnosis, assessment of prognosis, measurement of tumor burden and detection of minimal residual disease, early detection of disease recurrence, predicting or monitoring treatment response and detection of treatment resistance (Crowley et al., 2013; Buder et al., 2016; Mari et al., 2019; Vasconcelos et al., 2019). Most studies focus on blood-based biomarkers, however, urine, ascites, pleural effusion, and other biofluids can also be used (Lee J. S. et al., 2018; Mari et al., 2019).

Studies suggest that assessment of tumor-associated genetic changes in the blood could identify treatment resistance up to 10 months before radiological methods (Diaz et al., 2012; Misale et al., 2012). Liquid biopsy approach would therefore enable clinicians to modify or add treatment to achieve better response (Crowley et al., 2013). To date, most studies focused on identifying cancer-related genetic changes in CTCs or ctDNA. CTCs are intact tumor cells that actively or passively shed from primary or metastatic tumor into the bloodstream (Alimirzaie et al., 2019). Still, CTCs are rare events severely outnumbered by blood cells (1 per 1 ×10^6^–1 × 10^9^), resulting in limitations in CTC isolation and detection techniques and consequently low reproducibility of CTC-based tests (Geeurickx and Hendrix, 2020). Cell-free DNA (cfDNA) is fragmented DNA released from both healthy and tumor tissues from cells undergoing apoptosis or necrosis. ctDNA represents a subpopulation of cfDNA originating from cancer cells (Crowley et al., 2013; Alimirzaie et al., 2019). As ctDNA is highly fragmented [90–150 base pairs (bp)] and masked by a high background of total cfDNA, screening for clinically relevant mutations is challenging (Geeurickx and Hendrix, 2020). Currently, only five tests are Food and Drug Administration (FDA) approved, one CTC-based (CellSearch, Menarini Silicon Biosystems) and four cfDNA-based tests (cobas EGFR Mutation Test v2, Roche Molecular Systems; Epi proColon, Epigenomics; FoundationOne Liquid CDx, Foundation Medicine; Guardant360 CDx, Guardant Health). However, limited sensitivity or specificity as well as technological and regulatory challenges prevent a more widespread use in standard clinical practice (Vasconcelos et al., 2019).

**FIGURE 1:**
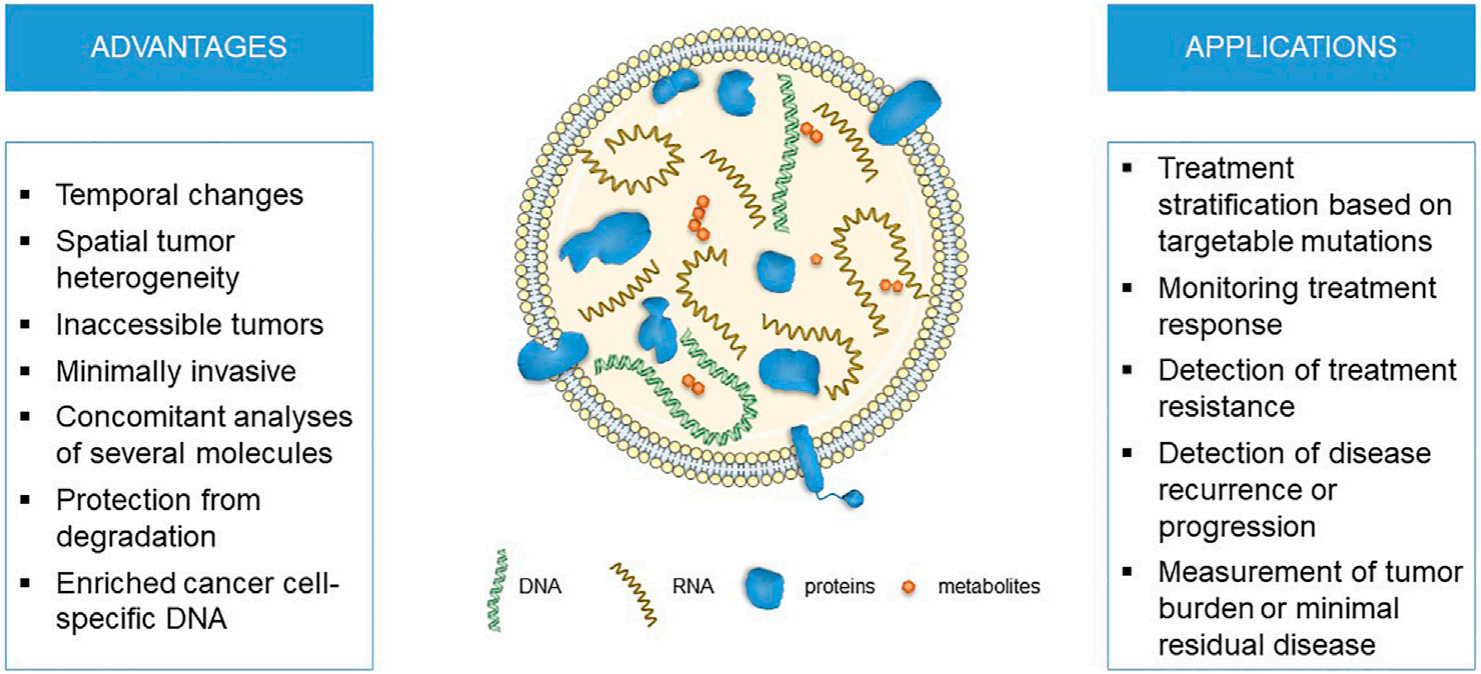
The advantages and applications for liquid biopsies using extracellular vesicle DNA in cancer personalized medicine and pharmacogenomics.

In recent years, EVs emerged as a novel analyte used for liquid biopsies. In this review, we will focus on EVs as novel source of biomarkers for personalized medicine and pharmacogenomics in oncology (Figure 1). As several genes associated with targeted therapy, chemotherapy or hormonal therapy were already detected in EVs, they might be used for fine-tuning personalized cancer treatment.

## Extracellular Vesicles

EVs are a heterogeneous population of membrane bound particles, which are released from all cells and accumulate into various body fluids *in vivo* (Lee J. S. et al., 2018; Dhondt et al., 2018; Hur et al., 2018; Johnsen et al., 2019). According to their size and site of formation, EVs can be broadly divided into three main categories: exosomes, microvesicles and apoptotic bodies. Exosomes range from 30 to 150 nm in diameter and are formed as intraluminal vesicles in multivesicular bodies, which fuse with plasma membrane to release them extracellularly. Microvesicles are larger membrane structures, from 100 to 1,000 nm in diameter, that are formed directly at the plasma membrane by outward budding. If they are released from tumors and transport tumor molecular cargo (e.g., oncoproteins), they are referred to as oncosomes, which were reported to reach up to 10 µm in diameter (Vagner et al., 2018). In contrast to exosomes and microvesicles, apoptotic bodies (up to 5 um in diameter) are formed during late phase of programmed cell death by membrane blebbing. Cells can simultaneously use more than one pathway of EV formation, which can be rerouted during pathogenic processes (Colombo et al., 2014; van Niel et al., 2018). Due to this complexity, EV subcellular origin is difficult to establish, and it is therefore preferred to use the terms small EVs and larger EVs for EVs <200 and >200 nm in size, respectively, (Théry et al., 2018).

Increase in plasma EV levels and changes in EV size were observed in several cancers (Silva et al., 2012; Muller et al., 2015; Huang et al., 2018; Navarro et al., 2019; Badovinac et al., 2021). To at least some extent, this is due to present tumor mass (Lai et al., 2017; Osti et al., 2019; Rodríguez Zorrilla et al., 2019), but systemic response to disease like inflammation (Roxburgh and McMillan, 2014; Chan et al., 2019) and response to treatment (Stevic et al., 2020) could also contribute. Increase in plasma EV levels is also observed in other diseases such as cardiovascular and autoimmune (Dickhout and Koenen, 2018; Maione et al., 2020) and might be connected to common physiological factors like hypoxia, autophagy or stress that are also often altered in tumors (Vasconcelos et al., 2019). Importantly, the pathological state of cell of origin additionally affects molecular composition of released EVs (van Niel et al., 2018). In general, EVs consist of a lipid bilayer membrane that surrounds a small amount of cytosol, and they contain various typical proteins [proteins involved in membrane trafficking, tetraspanins, adhesion molecules, cytoskeletal proteins, endosomal proteins (Kowal et al., 2016; Zhang H. et al., 2018; Théry et al., 2018)], lipids [ceramide, cholesterol, phosphatidylserine (Skotland et al., 2020)], nucleic acids [miRNA, mRNA, and DNA (Elzanowska et al., 2020; O'Brien et al., 2020)] and metabolites (Puhka et al., 2017). In cancer patients, EVs in body fluids were shown to accumulate oncogenes, tumor suppressor genes and their products, signature proteins and RNAs, and mutated genomic DNA (Zocco et al., 2014; An et al., 2015; González and Falcón-Pérez, 2015; Rowland et al., 2019; Vasconcelos et al., 2019; Chennakrishnaiah et al., 2020).

DNA only recently gained attention as an important constituent of EVs or other extracellular particles and remains poorly understood. EV-DNA varies in its localization and structure (Elzanowska et al., 2020; Malkin and Bratman, 2020). It is attached to the surface or is present in the lumen of EVs and can range in size from 200 bp in small EVs to up to >2 million bp in large EVs. EV-DNA exists as single or double stranded molecule of genomic or mitochondrial origin, mostly protected from degradation by bound histones. Reported heterogeneity might reflect diversity in EV biogenesis, as DNA content was shown to differ between EV subtypes (Lázaro-Ibáñez et al., 2014; Vagner et al., 2018; Lázaro-Ibáñez et al., 2019). EV-DNA is actively secreted from living cells (in contrast to passive secretion via necrosis), but little is known of the exact mechanisms. Those might include sequestering of cytosolic DNA during outward plasma membrane budding or intraluminal vesicles formation, or shuttling of collapsed micronuclei (small buddings of nucleus) to multivesicular bodies (Elzanowska et al., 2020; Malkin and Bratman, 2020). The latter mechanism is supported by observations of increased micronuclei production and EV-DNA release after genotoxic drug exposure (Yokoi et al., 2019). The biological function of EV-DNA is poorly understood, but the few reported studies propose their role i) in cellular homeostasis, as excretion of damaged DNA might prevent induction of apoptosis (Takahashi et al., 2017), ii) in transfer of genetic material, as recipient cells might transcribe or integrate DNA into the genome (Cai et al., 2013; Sansone et al., 2017), and iii) in immune response as it stimulates pro-inflammatory signaling pathways or anti-tumor immunity (Kitai et al., 2017; Torralba et al., 2018). Importantly, EV-DNA is protected from degradation in biofluids (Jin et al., 2016) and was shown to represent the entire genome and mutational status of the cell of origin (Kahlert et al., 2014; Thakur et al., 2014; San Lucas et al., 2016; Lázaro-Ibáñez et al., 2019).

EVs are important agents of inter-cellular communication that were implicated in numerous physiological and pathological processes. They were shown to cross extracellular space and even biological barriers, to interact with and/or transfer molecular content of the target cells, thereby affecting their cellular processes (Alvarez-Erviti et al., 2011; Colombo et al., 2014; van Niel et al., 2018). In cancer, EVs can act locally to remodel the tumor microenvironment at primary sites [reviewed in (Vader et al., 2014; Clancy and D'Souza-Schorey, 2018; Nogués et al., 2018)]. EVs (oncosomes) are released from tumor cells and transport bioactive cargo to neighboring tumor cells, stromal cells and cells of the immune system, thereby promoting angiogenesis, immunosuppression, tumor growth and local invasion. Alternatively, EVs accumulate in diverse body fluids (e.g., blood, lymphatic fluid) and are transported to local (e.g., lymph node) or distant sites of metastasis (e.g., lung, brain, bone, liver), depending on EV-organotropism. There, they promote pre-metastatic niche formation by inducing angiogenesis, remodeling of stroma and extracellular matrix, and by modulating immune responses.

EVs traceability of their cellular origin and its (patho)physiological state, high concentrations in body fluids and their ability to protect molecular cargo from degradation makes them a promising source of minimally invasive biomarkers. This is especially true in cancer, as EVs dynamically reflect changes in tumor molecular profiles depending on disease progression and treatment effectiveness (An et al., 2015; Syn et al., 2016; Rofi et al., 2019; Vasconcelos et al., 2019; Chennakrishnaiah et al., 2020; LeBleu and Kalluri, 2020). Compared to other liquid biopsy analytes, EVs represent multicomponent biomarker platforms that enable concomitant analyses of several molecules carried in the same vesicles (LeBleu and Kalluri, 2020). EVs are more abundant in various biofluids and thus could better represent intra-tumor heterogeneity than CTCs and also reflect stromal response. They protect their molecular cargo from degradation, may enrich for cancer cell-specific DNA and provide better signal-to-noize ratio compared to ctDNA (Vasconcelos et al., 2019; LeBleu and Kalluri, 2020). EVs could also help monitor variability in host responses through immune, epithelial or endothelial-derived EVs (LeBleu and Kalluri, 2020). Altogether, diagnostics based on EVs could enable a more comprehensive assessment of cancer diagnosis, prognosis and progression, which could guide a more personalized and effective treatment of cancer patients.

Importantly, EVs also contribute to transfer of oncogenic potential or drug resistance during chemotherapy. For example, in breast cancer treated with trastuzumab, EV-specific proteins were upregulated in metastatic patients that benefited from therapy, but not in those where treatment failed (Drucker et al., 2020). Alternatively, neoadjuvant breast cancer therapies with taxanes and anthracyclines induced tumor-derived EVs with enhanced pro-metastatic capacity (Keklikoglou et al., 2019). On the other hand, tumor EVs can spread drug resistance to drug sensitive cells. Systemic temozolomide treatment in glioblastoma mouse models induced mRNA expression profile reflective of drug resistance, which was recapitulated in the transcriptome of released EVs (Garnier et al., 2018). In oxidative phosphorylation-dependent breast cancer, horizontal transfer of EV-bound mitochondrial DNA promoted exit from dormancy of therapy-induced cancer stem-like cells and lead to endocrine therapy resistance (Sansone et al., 2017). Additionally, EVs were implicated in chemotherapy drug (doxorubicin, cisplatin, cetuximab, pixantrone) efflux in *vitro* experiments (Shedden et al., 2003; Safaei et al., 2005; Koch et al., 2016; Fujiwara et al., 2018), a possible mechanism for reducing their effective concentration at target sites. Another study showed that malignant lymphoma EVs carried CD20, which bound therapeutic anti-CD20 antibodies and protected target cells from antibody attack (Aung et al., 2011). Comprehensive overviews of experimental data on EVs relevance in drug resistance are provided in reviews by Vasconcelos et al. (Vasconcelos et al., 2019) and Maacha et al. (Maacha et al., 2019).

A limited number of clinical studies investigated EVs to monitor cancer progression, tumor evolution or drug resistance and several putative protein and RNA biomarkers were identified (An et al., 2015; González and Falcón-Pérez, 2015; Dhondt et al., 2018; Vasconcelos et al., 2019). For example, GPC1-positive EVs were shown to successfully diagnose pancreatic ductal adenocarcinoma (PDAC) and correlated with overall survival (Melo et al., 2015), while EpCAM-positive EVs could predict progression free survival (Giampieri et al., 2019). In PDAC patients treated with gemcitabine, high expression of miRNA-155 in tumor tissue and plasma EVs correlated with poorer prognosis due to chemoresistance development (Mikamori et al., 2017). In breast cancer, levels of TRPC5-positive EVs negatively correlated with chemotherapy outcome and could predict progression free survival (Wang et al., 2017). Additionally, levels of EV-associated lncRNA HOTAIR were associated with tumor burden and aggressiveness of the disease (Tang et al., 2019). Specific EV-miRNAs were also shown to correlate with tumor burden and response to therapy in breast cancer (Rodríguez-Martínez et al., 2019). EV-associated UCH-L1 mRNA was associated with ER-/PR-aggressive breast cancers subtypes and poor prognosis (Miyoshi et al., 2006), and with poor response to adjuvant anthracycline/taxane-based chemotherapy (Ning et al., 2017). HER2-positive breast cancer patients that did not respond to treatment had higher levels of EV lncRNA SNHG14 (Dong et al., 2018) or TGF-β1 (Martinez et al., 2017). Specific EV-associated non-coding RNAs were also predictive of treatment response to gemcitabine (Wei et al., 2017), erlotinib (Zhang W. et al., 2018) or cisplatin (Yuwen et al., 2017) in non-small cell lung cancer (NSCLC). Interestingly, EVs and their cargo were also associated with immunosuppression and response to immunotherapy with immune checkpoint inhibitors (Chen et al., 2018). In melanoma, EVs carrying immune check-point ligand PD-L1 on their surface, captured the therapeutic anti-PD-1 antibodies and drove antibody away from target tumor (Chen et al., 2018). Temporal changes in EV-associated PD-L1 mRNA levels were associated with response to anti-PD-1 antibodies nivolumab and pembrolizumab in patients with melanoma and NSCLC (Del Re et al., 2018). This suggests EV liquid biopsy could be implemented also in other areas of cancer treatment. Key biomarkers implicated in cancer personalized medicine and pharmacogenomics are described in *Extracellular Vesicles and Cancer Personalized Medicine* and *Extracellular Vesicles and Cancer Pharmacogenomics*.

## Extracellular Vesicles and Cancer Personalized Medicine

In recent years, targetable mutations and personalized medicine have significantly affected treatment options and outcomes for cancer patients, especially in solid tumors (Rofi et al., 2019). Targeted cancer treatment refers to the use of drugs that target specific molecules in tumor cells or tumor microenvironment involved in signaling, cell survival and proliferation to prevent the growth and spread of tumors (Lee Y. T. et al., 2018). Targeted drugs can be small molecules or monoclonal antibodies. Small molecules are usually tyrosine kinase inhibitors, proteasome inhibitors, cyclin-dependent kinase inhibitors or poly ADP-ribose polymerase inhibitors (Lee Y. T. et al., 2018). Monoclonal antibodies usually directly or indirectly interrupt key signaling pathways (Lee Y. T. et al., 2018). Numerous drugs are used for targeted treatment across several cancer types (Table 1). However, treatment toxicity and inherent or acquired resistance to therapy pose a challenge for monitoring patients and their disease and better biomarkers or tumor molecular profiling strategies are still needed (Panagiotara et al., 2017). Currently, individual genetic alterations are used to guide treatment selection (Crowley et al., 2013). As targeted treatment is often used in advanced tumors, determination of genetic profile using tissue biopsy samples obtained at diagnosis is not optimal as they do not reflect the state of the tumor at the time of the treatment. Liquid biopsies can be used to identify acquired mutations or resistance that would allow the selection of the most efficient treatment (Crowley et al., 2013).

For this review, cancer genome very important pharmacogenes (VIPs), required pharmacogenetic testing, and pharmacogenetic recommendations available until June 1^st^ 2020 were extracted from PharmGKB (Whirl-Carrillo et al., 2012). Data on the association of the selected human genes with extracellular vesicles was compiled using ExoCarta (Keerthikumar et al., 2016) and Vesiclepedia (Kalra et al., 2012; Pathan et al., 2019) databases that represent manually curated web-based compendiums of EV proteins, RNAs and lipids. Additional literature was selected using PubMed based on the following searches: “extracellular vesicles and gene name” or “exosomes and gene name”. Data from animal studies was not included.

According to PharmGKB (Whirl-Carrillo et al., 2012), nine VIPs are associated with cancer genome and personalized medicine. Genetic testing based on these genes is required by United States FDA and/or European Medicines Agency (EMA) for 40 different target drugs (Table 1). All cancer VIPs were already identified in EVs, usually both on protein and mRNA level. Additionally, EV-DNA could be used in liquid biopsies predicting or monitoring response to their target drugs by monitoring the specific landscape of somatic mutations. A more detailed description of VIPs related to their functional and biomarker role as part of EVs is provided in chapters 3.1 through 3.7.

### ALK

ALK is a receptor tyrosine kinase important for the development of the nervous system (Cesi et al., 2018). Genetic rearrangements or mutations can lead to its constitutive activation and activation of downstream signaling pathways that affect proliferation and apoptosis in other differentiated tissues (Cesi et al., 2018; Wu et al., 2018). *ALK* therefore functions as an oncogene in several cancers, including NSCLC and neuroblastoma (Cesi et al., 2018). *ALK* fusion occurs in 3–7% of lung cancers, most commonly with *EML4* (Chen et al., 2017). ALK inhibitors (e.g., crizotinib) improved treatment response and survival in patients with *ALK* rearrangements, but acquired treatment resistance is frequently a problem (Chen et al., 2017). Real-time monitoring using liquid biopsy could be used to detect the emergence of resistant mutations, however, there is limited knowledge about the connection of *ALK* with EVs (Table 1) (Chen et al., 2017). Irradiation of ALK-positive NSCLC cells lead to increased expression of ALK in EVs that were able to activate signaling pathways in recipient cells (Wu et al., 2018). In a pilot study, *EML4-ALK* translocation was detected in plasma-derived EVs of patients with NSCLC, suggesting that EVs may serve as an additional tool to guide treatment with ALK inhibitors (Reclusa et al., 2019).

### BCR-ABL1

ABL1 is a proto-oncogene tyrosine kinase involved in cell differentiation, division, and proliferation. The role of BCR in not completely understood, but it has serine/threonine kinase activity and is a GTPase-activating protein. *BCR*-*ABL1* fusion known as the Philadelphia chromosome that occurs due to a reciprocal translocation between chromosomes 9 and 22 is the driver fusion in chronic myeloid leukemia (CML) (Fu et al., 2017; Jurj et al., 2020). The fusion leads to dysregulation and over-activation of ABL1 tyrosine kinase domain. Several tyrosine kinase inhibitors such as imatinib target the fusion protein and have significantly improved survival of CML patients (Fu et al., 2017; Jurj et al., 2020). On the other hand, several mutations in *ABL1* gene were associated with imatinib resistance. BCR, ABL1 and *BCR*-*ABL1* fusion were frequently detected in EVs (Table 1). EV transfer of the oncogenic *BCR*-*ABL1* transcript was able to affect signaling in mesenchymal stem cells and increase their proliferation (Zhu et al., 2014; Fu et al., 2017; Milani et al., 2017). *BCR*-*ABL1* expression was already detected in serum-derived EVs of CML patients (Fu et al., 2017; Kang et al., 2018) and was associated also with response to tyrosine kinase inhibitors, imatinib resistance and disease remission (Kang et al., 2018; Liu et al., 2018; Jurj et al., 2020). EVs were also proposed as a novel biomarker for monitoring minimal residual disease through the number of *BCR*-*ABL1* copies (Fu et al., 2017; Jurj et al., 2020).

### BRAF

*BRAF* encodes a serine/threonine-specific mitogen-activated protein kinase (MAPK) kinase, which is part of the MAPK/ERK signaling cascade regulating cell proliferation. Presence of growth factors activates target membrane receptors, which in turn activate Ras GTPases that recruit BRAF to the membrane and induce homo or hetero-dimerization and activation of downstream ERK (Brummer and McInnes, 2020). *BRAF* alterations are important drivers of human cancer, particularly in melanoma (found in 40–50%), thyroid cancers (10–70%), colorectal cancers (10%) and NSCLC (3–5%) (Dankner et al., 2018). The most studied BRAF V600 allele functions as a hyperactive monomer, uncoupled from upstream regulation by Ras GTPase, thereby promoting constitutive activation of cell growth and proliferation (Dankner et al., 2018). BRAF inhibitors (e.g., dabrafenib and vemurafenib) alone or combined with MEK inhibitors provide a significant survival benefit, but over time tumors develop acquired resistance. Some BRAF V600 and non-V600 tumors also show intrinsic resistance, are insensitive to inhibitors or even cause paradoxical activation of ERK (Dankner et al., 2018). Further liquid biopsy biomarkers are needed to better guide targeted therapies for BRAF mutant cancers. EVs could serve as a novel source for genotyping *BRAF* or as biomarker of disease progression, since V600 mutation was detected in cancer cell line- and in body fluid-derived EVs in various cancers (Table 1), and corresponded to disease state, prognosis or survival (Hong et al., 2009; Thakur et al., 2014; Hao et al., 2017; Klump et al., 2018; García-Silva et al., 2019; Pasqualetti et al., 2019; Song et al., 2019).

### EGFR

*EGFR* (also known as *ERBB1*) encodes a member of the epidermal growth factor receptor (EGFR) family of receptor tyrosine kinases, which regulate epithelial cell survival, proliferation, differentiation, and motility. Binding of one of its seven ligands (e.g., EGF and TGFα) promotes EGFR homo- or hetero-dimerization, which through structural rearrangements promotes *trans*-autophosphorylation of the cytosolic tyrosine kinase domains and activation of several signaling pathways including the Ras/MAPK, PI3K/AKT and PLC/PKC (Sigismund et al., 2018). *EGFR* mutations, frequently combined with overexpression, are important drivers of human cancers, particularly in glioblastoma (found in 50%), NSCLC (15–20%) and colorectal cancers (3%). Oncogenic EGFR (e.g., EGFRvIII, L858R) stimulate receptor homo- and heterodimerization and abnormal EGFR endocytic trafficking, which contributes to increased kinase activation and downstream signaling (Sebastian et al., 2006; Sigismund et al., 2018). Currently, monoclonal humanized antibodies (cetuximab, panitumumab) or tyrosine kinase inhibitors (e.g., erlotinib) targeting oncogenic EGFR show limited response and frequently evoke resistance in patients (Sebastian et al., 2006; Sigismund et al., 2018). Liquid biopsy, more specifically EVs, could contribute to improved cancer progression and therapy follow-up, as EVs containing EGFR DNA, RNA or protein were detected in blood, cerebrospinal fluid, bronchoalveolar lavage or pleural effusions in different cancers (Table 1), which in some studies correlated with prognosis (Skog et al., 2008; Shao et al., 2012; Yamashita et al., 2013; Figueroa et al., 2017; Lee J. S. et al., 2018; Castellanos-Rizaldos et al., 2018; Krug et al., 2018; Wan et al., 2018; Ortega et al., 2019; Qu et al., 2019; Song et al., 2019). For example, anticancer agents blocking oncogenic EGFR (e.g., dacomitinib, canertinib) stimulated the release of EVs carrying EGFRvIII and genomic DNA in glioblastoma animal models and patients (Skog et al., 2008; Montermini et al., 2015; Choi et al., 2019), and in turn, EV-EGFRvIII were shown to fuse with cancer cells lacking EGFRvIII and transfer the oncogenic activity (Al-Nedawi et al., 2008). EV-bound DNA was superior to cfDNA for *EGFR* mutation detection in early stage NSCLC (Wan et al., 2018). In lung adenocarcinoma, mutation status of EV-associated DNA from pleural effusion correlated with cfDNA (Song et al., 2019), while presence of mutated *EGFR* informed about the EGFR tyrosine kinase inhibitor treatment efficacy (Qu et al., 2019). EGFR EVs release additionally responded to cetuximab, TKIs or temozolomide treatment of cells *in vitro* (Shao et al., 2012; Montermini et al., 2015; van Dommelen et al., 2016; Fujiwara et al., 2018). *In vitro*, EGFR EVs were shown to be involved in several cancer hallmarks like inducing angiogenesis, sustaining proliferating signaling, evading growth suppression and immune destruction, promoting inflammation, resisting cell death, and activating invasion and metastasis (reviewed in (Zanetti-Domingues et al., 2020)). Further studies on EVs as therapy delivery system are also needed, as EVs containing let-7a reduced tumor size in mice EGFR-expressing breast cancer model (Ohno et al., 2013).

### ERBB2

*ERBB2* encodes a receptor tyrosine kinase also known as HER2 that belongs to EGFR family. Presence of growth factors leads to heterodimerization and activation of MAPK and PI3K signaling pathways (Graus-Porta et al., 1997). *ERBB2* is a proto-oncogene that is overexpressed in approximately 25% of breast cancers, but also in gastric and other cancers, leading to malignant transformation of epithelial cells (Slamon et al., 2011; Fang et al., 2017; Galli de Amorim et al., 2019). Overexpression occurs mostly due to gene amplification, but other activating mutations were also described (Slamon et al., 2011; Galli de Amorim et al., 2019). *ERBB2* overexpression is associated with a more aggressive phenotype with worse survival because of increased proliferation, invasiveness and metastasis (Slamon et al., 2011). On the other hand, ERBB2 represents a target for several different drugs, both monoclonal antibodies (e.g., trastuzumab) and tyrosine kinase inhibitors (e.g., lapatinib and neratinib). Introduction of targeted treatment significantly improved response and survival in HER2-positive breast cancer patients (Slamon et al., 2011). However, not all patients respond to treatment with trastuzumab and some patients develop resistance, therefore additional biomarkers are needed to further improve treatment outcome (Ciravolo et al., 2012; Galli de Amorim et al., 2019). ERBB2 was frequently identified in EVs (Table 1). *ERBB2* expression was increased in EVs from HER2-positive cell lines or in blood-derived EVs of cancer patients (Baran et al., 2010; Ciravolo et al., 2012; Wang et al., 2018). Studies have shown that ERBB2 expression in blood or urine-derived EVs correlates with tissue expression and liquid biopsy may sometimes even be more sensitive to detect ERBB2 overexpression (Fang et al., 2017; Lee D. H. et al., 2018; Platko et al., 2019). Additionally, ERBB2 overexpression alters EV protein composition (Amorim et al., 2014). Studies also suggest activated ERBB2 in EVs can sequester trastuzumab, potentially affecting its efficacy (Ciravolo et al., 2012). EVs could therefore serve as an additional biomarker for personalized treatment of HER2-positive cancer patients.

### KIT

KIT is a proto-oncogene receptor tyrosine kinase that influences cell growth, survival, proliferation, differentiation and migration through the activation of downstream signaling pathways (Wu et al., 2019). Activating *KIT* mutations are present in 75–85% of gastrointestinal stromal tumors (GISTs), representing the main oncogenic driver (von Mehren and Joensuu, 2018; Wu et al., 2019). Up to 90% of *KIT* mutations occur in exon 11, making cells sensitive to tyrosine kinase inhibitor imatinib (von Mehren and Joensuu, 2018; Wu et al., 2019). However, secondary mutations in other exons can lead to imatinib resistance (Wu et al., 2019). KIT was already detected in various cancer cell line EVs (Table 1). GIST cells were shown to secrete a lot of oncogenic EVs containing KIT. Uptake of these EVs by stromal cells lead to their conversion to tumor promoting cells, activation of signaling pathways, induction of MMP1 expression and increased tumor cell invasion (Atay et al., 2014). However, the data on monitoring *KIT* mutations in EVs is lacking. In one study that included only three patients, one *KIT* mutation associated with treatment resistance was only present in cfDNA (Klump et al., 2018). Further studies are needed to determine the usefulness of *KIT* mutations detection in EVs.

### RAS (KRAS, NRAS)

*KRAS* and *NRAS* genes encode small membrane-localized GTPases, important for regulating cell growth, differentiation and survival. When growth factors bind target cellular receptors (e.g., receptor tyrosine kinases, G-protein coupled receptors, integrins), they activate RAS through recruitment of scaffolding proteins that mediate conversion of inactive GDP-bound to active GTP-bound form, which in turn activates diverse MAPK pathways. RAS activity can be further regulated by various proteins and post-translational modifications (e.g., farnesylation, palmitoylation, ubiquitination, acetylation) (Gimple and Wang, 2019). RAS alterations are drivers of 20–30% of all human cancers. *KRAS* mutations (mostly on the 12^th^ codon) are exceedingly common in pancreatic adenocarcinomas (found in 94%), colorectal cancers (37%) and lung adenocarcinomas (20–30%), while *NRAS* mutations (mostly on 61^st^ codon) are more common in melanomas (20–30%), thyroid cancers (10–40%, varies among different types), and leukemias (15%) (Cerami et al., 2012; Gao et al., 2013). They enhance RAS activity, effectively uncoupling downstream signaling from growth factor receptors, thereby promoting tumor cell proliferation, survival, metabolism, microenvironmental interactions and immune evasion (Gimple and Wang, 2019). RAS is believed to be an undruggable target due to its molecular structure, but RAS signaling can be indirectly targeted through its regulators (e.g., cetuximab) or downstream effectors (e.g., vemurafenib). Still, development of therapeutic resistance is common and indirect targeting can cause counterproductive effects (e.g., BRAF inhibitors) (Crowley et al., 2013; Gimple and Wang, 2019). Importantly, mutant KRAS was shown to affect composition of EVs released by cancer cells and its DNA and protein molecules were detected in EVs from blood and pleural effusion of different cancers (Table 1) (Demory Beckler et al., 2013; Park et al., 2013; Kahlert et al., 2014; Lee et al., 2014; Yang S. et al., 2017; Möhrmann et al., 2018). Level of mutant KRAS EVs in plasma even correlated with response to therapy and tumor burden with pancreatic cancer (Kahlert et al., 2014; Allenson et al., 2017; Yang S. et al., 2017; Bernard et al., 2019). Oncogenic NRAS was detected in EVs from various cell lines and similarly released by EVs from mouse brain tumor cells (Lee et al., 2014). When tested *in vitro* for function, mutant KRAS EVs stimulated cell proliferation, altered metabolic state and enhanced invasiveness of recipient cells (Demory Beckler et al., 2013; Lee et al., 2014; Zhang Q. et al., 2018). Thus mutant KRAS EVs could be used for rapid, non-invasive and continuous identification of cancer driver mutations, relevant for personalized treatment strategies. Further studies on EVs as therapy delivery system are also needed, as EVs containing siRNA/shRNA against oncogenic KRAS suppressed cancer and increased overall survival in multiple mouse models of pancreatic cancer (Kamerkar et al., 2017).

## Extracellular Vesicles and Cancer Pharmacogenomics

Among key pharmacogenes are drug metabolizing enzymes such as cytochromes P450 (CYP) and UDP-glucuronosyltransreases (UGT) and ATP-binding cassette (ABC) transporters. CYPs and UGTs are involved in metabolic clearance of more than 90% of drugs (Rowland et al., 2019). Activity of CYPs and UGTs is influenced by their function and expression. Pharmacogenetics studies have found several genetic variants that modify enzyme activity (Rodriguez-Antona and Ingelman-Sundberg, 2006), however, genetic factors are often not sufficient to predict patient drug exposure (Rowland et al., 2019). Recent evidence suggests several pharmacogenes are also present in EVs (Kumar et al., 2017; Gerth et al., 2019; Rowland et al., 2019). For example, EVs isolated from human plasma contained functional proteins and mRNAs of different CYPs and UGTs (Kumar et al., 2017; Rowland et al., 2019). Due to relatively high abundance and confirmed enzymatic activity, circulating EV CYPs may also have a physiological role in extrahepatic drug metabolism (Kumar et al., 2017). For example, CYP2E1 is highly expressed in plasma EVs and was also associated with exacerbating alcohol and acetaminophen-induced toxicity in hepatic and extrahepatic cells (Kumar et al., 2017; Rahman et al., 2019).

CYPs play an important role in the activation or inactivation of several cancer drugs (Rodriguez-Antona and Ingelman-Sundberg, 2006). For example, one of the most important enzymes, CYP3A4, is involved in metabolism of docetaxel, etoposide, cyclophosphamide, vincristine, and paclitaxel (Rodriguez-Antona and Ingelman-Sundberg, 2006). Rare CYP3A4*22 and *1B alleles alter CYP3A4 activity, but genotyping cannot explain all interindividual differences in CYP3A4 expression or function and no drug prescribing recommendations are currently in use (Rowland et al., 2019). CYP3A4 activity was confirmed also in plasma EVs (Kumar et al., 2017; Rowland et al., 2019). Additionally, treatment with CYP3A4 inductor rifampicin significantly increased CYP3A4 expression in EVs and CYP3A4 expression in plasma EVs correlated with midazolam clearance (Rowland et al., 2019). These results suggest that measuring CYP3A4 expression in EVs could help predict response to drugs metabolized by this enzyme. However, the patients in published studies were selected for wild-type *CYP3A4* genotype and further studies evaluating EV expression of CYP3A4 are needed to evaluate the role of CYP3A4 from EVs. Combination of *CYP3A4* genotyping and liquid biopsy could potentially serve as a novel biomarker for identifying variability in drug exposure.

Several other pharmacogenes can influence response to cancer therapy and for some of them, drug prescribing recommendations (e.g., dose adjustment, alternative drugs) are already available (Table 2). According to PharmGKB (Whirl-Carrillo et al., 2012), four cancer pharmacogenes have pharmacogenetic drug prescribing recommendations from Clinical Pharmacogenetics Implementation Consortium (CPIC) or Dutch Pharmacogenetics Working Group (DPWG) (PharmGKB level 1A and 1B clinical annotations). Additional six genes are also important for predicting response or adverse events to chemotherapy (PharmGKB level 2A clinical annotations). Among these ten genes, all except *SLC O 1B1* were previously identified in EVs. Most genes were detected in EVs from different cancer cell lines (protein and/or mRNA), several were also detected in urine. A more detailed evaluation of the connection with EVs is described for UGT1A1, ABCB1, and GSTP1.

**TABLE 2:** List of pharmacogenes associated with cancer therapy and the evidence connecting them with extracellular vesicles (EVs).

Gene	Drug	Therapy type	Connection with extracellular vesicles: Examples	References^a^
Genes with pharmacogenetic drug prescribing recommendations^b^				
*CYP2D6*	Tamoxifen	Hormone therapy	Protein detected in urine EVs (healthy donors, patients with nephropathy)	Moon et al. (2011)
*DPYD*	Capecitabine, fluorouracil, tegafur	Chemotherapy	Protein detected in glioblastoma cells EVs and in urine EVs (healthy donors)	Fraser et al. (2013), Pavlyukov et al. (2018)
*TPMT*	Mercaptopurine, thioguanine	Chemotherapy	Protein detected in various cancer cell line EVs and in urine EVs (healthy donors)	Hong et al. (2009), Wang et al. (2012), Bijnsdorp et al. (2013), Liang et al. (2013), Sinha et al. (2014), Liu et al. (2015), Hurwitz et al. (2016), Liem et al. (2017)
			mRNA detected in colorectal cancer cell EVs	
*UGT1A1*	Irinotecan	Chemotherapy	Protein detected in platelet EVs	Dean et al. (2009), Liu et al. (2020)
			mRNA detected in plasma derived EVs in lung adenocarcinoma	
Genes with PharmGKB level 2A clinical annotations				
*ABCB1*	Methotrexate	Chemotherapy	Protein detected in various cancer cell line EVs, platelet EVs, serum EVs in prostate cancer and urine EVs (healthy donors)	Dean et al. (2009), Gonzales et al. (2009), Hong et al. (2009), Kato et al. (2015), Kharaziha et al. (2015), Hurwitz et al. (2016), Wang et al. (2019)
			mRNA detected in colorectal cancer cell EVs	
*NQ O 1*	Alkylating agents, anthracyclines and related substances, fluorouracil, platinum compounds	Chemotherapy	Protein detected in various cancer cell line EVs and in urine EVs (healthy donors)	Hong et al. (2009), Welton et al. (2010), Wang et al. (2012), Ji et al. (2013), Hurwitz et al. (2016), Liem et al. (2017), Choi et al. (2018)
			mRNA detected in colorectal cancer cell EVs	
*MTHFR*	Carboplatin, cisplatin, methotrexate	Chemotherapy	Protein detected in various cancer cell line EVs	Hong et al. (2009), Hurwitz et al. (2016), Liem et al. (2017)
			mRNA detected in colorectal cancer cell EVs	
*GSTP1*	Fluorouracil, oxaliplatin, cyclophosphamide, epirubicin, platinum compounds	Chemotherapy	Protein detected in various cancer cell line EVs and in urine EVs (healthy donors, prostate cancer)	Skog et al. (2008), Gonzales et al. (2009), Hong et al. (2009), Welton et al. (2010), Fraser et al. (2013), Ji et al. (2013), Paggetti et al. (2015), Øverbye et al. (2015), Hurwitz et al. (2016), Yang S. et al. (2017), Lázaro-Ibáñez et al. (2017), Liem et al. (2017), Choi et al. (2018), Zhang et al. (2019)
			mRNA detected in colorectal cancer and glioblastoma cell EVs and serum derived EVs in breast cancer patients	
*SLCO1B1*	Methotrexate	Chemotherapy	Not reported	—
*TYMS*	Capecitabine, fluorouracil	Chemotherapy	Protein detected in various cancer cell line EVs	Skog et al. (2008), Hong et al. (2009), Paggetti et al. (2015), Hurwitz et al. (2016), Liem et al. (2017), Choi et al. (2018)
			mRNA detected in colorectal cancer and glioblastoma cell EVs	

a References from individual functional/biomarker EV studies and EV-omics studies from VesiclePedia or Exocarta.

b Pharmacogenetic recommendations from CPIC or DPWG reported in PharmGKB, level 1A and 1B clinical annotations.

### UGT1A1

Among pharmacogenes with drug prescribing recommendations, only uridine diphosphate glucuronosyltransferase 1A (UGT1A1) was already assessed as a potential biomarker in EVs (Table 2) (Liu et al., 2020). UGT1A1 catalyzes the glucuronidation of bilirubin and various xenobiotics, which enables their elimination (de Man et al., 2018). UGT1A1 is also involved in inactivation of SN-38, the active metabolite of irinotecan, a chemotherapeutic drug used for treatment of lung, colon, gastric, pancreatic, and gynecological cancers (Campbell et al., 2017; de Man et al., 2018). UGT1A1 is a very polymorphic enzyme, with over 100 genetic variants described (de Man et al., 2018). Most studied genetic variants are UGT1A1*6 and *28. Both are associated with increased irinotecan toxicity, especially neutropenia and diarrhea, due to increased systemic exposure to irinotecan and its metabolite (Campbell et al., 2017; de Man et al., 2018). For carriers of two polymorphic alleles, lower irinotecan starting dose is recommended (de Man et al., 2018). When comparing EVs from plasma of patients with lung adenocarcinoma and benign lung diseases using RNA-Seq, mRNA expression of *UGT1A1* could differentiate between both groups and was suggested as an additional biomarker in NSCLC patients (Liu et al., 2020).

### ABCB1

ABC transporter ABCB1, also known as multidrug resistance protein 1 (MDR1) or P-glycoprotein, is one of the key genes, involved in the development of chemoresistance to anticancer drugs (Kharaziha et al., 2015). ABCB1 is a cell surface glycoprotein that mediates ATP-dependent efflux of various molecules, including different xenobiotics (Gong et al., 2012; Kharaziha et al., 2015). Several *ABCB1* genetic variants have been described (e.g., rs1128503, rs2032582, and rs1045642), but due to inconsistent results, no drug prescribing recommendations are currently available (Whirl-Carrillo et al., 2012). However, rs1045642 was associated with methotrexate toxicity in lymphoma and leukemia (Suthandiram et al., 2014; Zgheib et al., 2014; Gregers et al., 2015). On the other hand, ABCB1 was consistently associated with intrinsic or acquired multidrug resistance and treatment failure in several cancer types (Gong et al., 2012). EVs were proposed as one of the mechanisms associated with drug resistance, as they can help efflux chemotherapeutic (e.g., cisplatin, doxorubicin) from tumor cells (Samuel et al., 2018; Gluszko et al., 2019). Additionally, EVs were involved in intercellular transfer of drug resistance from drug-resistant to sensitive cells via ABCB1 (Table 2) (Bebawy et al., 2009; Gong et al., 2012; Kato et al., 2015; Kharaziha et al., 2015; Maacha et al., 2019; Vasconcelos et al., 2019; Wang et al., 2019). ABCB1 was enriched in EVs from various drug-resistant cancer cells after chemotherapy (Wang et al., 2019) and was associated with acquired resistance to docetaxel (Kato et al., 2015; Kharaziha et al., 2015). ABCB1 expression was also increased in serum derived EVs from prostate cancer patients, resistant to docetaxel (Kato et al., 2015). Detection of ABCB1 expression in EVs with liquid biopsy could therefore serve as an important biomarker of cancer treatment resistance.

### GSTP1

Glutathione S-transferase pi 1 (GSTP1) is a phase II metabolic enzyme involved in the detoxification of various anti-cancer drugs and other carcinogens. GSTP1 catalyzes their conjugation with glutathione (Yang S.-J. et al., 2017). Two common non-synonymous polymorphisms, rs1695 and rs1138272, affect the electrophile-binding active site of the enzyme and alter enzyme activity (Ali-Osman et al., 1997). *GSTP1* genetic variability was associated with toxicity and efficacy of numerous chemotherapeutics, especially platinum compounds (Stoehlmacher et al., 2004; Lamas et al., 2011; Goricar et al., 2015). However, no drug prescribing recommendations are currently available due to inconsistent results among cancer types (Whirl-Carrillo et al., 2012; Campbell et al., 2016). GSTP1 was frequently identified in EVs (Table 2). GSTP1 expression was increased in EVs from 5-fluorouracil or adriamycin resistant cancer cell lines (Yang S.-J. et al., 2017; Zhang et al., 2019). Additionally, EV-mediated transfer of GSTP1 was associated with intercellular transfer of adriamycin resistance, which is also an important clinical problem (Yang S.-J. et al., 2017). In breast cancer patients treated with anthracycline/taxane chemotherapy, *GSTP1* mRNA expression was increased in serum EVs from patients with worse treatment response (Yang S.-J. et al., 2017). Detection of GSTP1 expression in EVs with liquid biopsy could therefore serve as an additional biomarker, potentially improving the predictive value of pharmacogenetics variants.

## Conclusion and Future Perspectives

EVs have a proven role in transfer of oncogenic potential or drug resistance during chemotherapy and were proposed as biomarkers of cancer progression, tumor evolution or drug resistance in several cancer types. Several pharmacogenes and genes associated with treatment response were already detected in EVs *in vitro* and *in vivo*, thus they might be used for fine-tuning personalized cancer treatment. Before implementation in clinical practice, technical challenges regarding standardization and repeatability of EV analysis and detection of rare genetic variants should be addressed. Additionally, sufficient sensitivity and specificity of the novel biomarker assays should be obtained. As genetic factors cannot account for all observed variability, composite biomarkers incorporating genetic factors and other EV cargo are needed for some drugs. This could further improve personalized treatment for various drugs used in targeted treatment and chemotherapy, especially drugs targeting inducible enzymes of enzymes with poor genotype-phenotype associations (Gerth et al., 2019; Rodrigues and Rowland, 2019). All biomarkers and methods for their determination also have to be validated in independent studies and approved by appropriate regulatory agencies (Novelli et al., 2010). Several genomic biomarkers have been recognized, but there are considerable differences between EMA and FDA recommendations in summary of product characteristics labels (Shekhani et al., 2020). Consensus among regulators would further contribute to clinical implementation of key biomarkers.

In conclusion, EVs are emerging as liquid biopsy analytes that have several advantages and could be used in various applications in oncology, from treatment stratification to detection of treatment response or resistance, enabling new possibilities in personalized medicine or pharmacogenomics.

## Abbreviations

ABC, ATP-binding cassette; bp, base pair; cfDNA, cell-free DNA; CML, chronic myeloid leukemia; CPOC, clinical pharmacogenetics implementation consortium; CTC, circulating tumor cell; ctDNA, circulating tumor DNA; ctRNA, circulating tumor RNA; CYP, cytochromes; DPWG, dutch pharmacogenetics working group; EGFR, epidermal growth factor receptor; EMA, European Medicines Agency; EV, extracellular vesicle; FDA, Food and Drug Administration; GIST, gastrointestinal stromal tumor; MAPK, mitogen-activated protein kinase; NSCLC, non-small cell lung cancer; PDAC, pancreatic ductal adenocarcinoma; UGT, UDP-glucuronosyltransrease; VIP, very important pharmacogene
